# Clinical impact of assessing thrombus age using magnetic resonance venography prior to catheter-directed thrombolysis

**DOI:** 10.1007/s00330-022-08599-5

**Published:** 2022-03-28

**Authors:** Carsten W. K. P. Arnoldussen, Pascale Notten, Rutger Brans, Dammis Vroegindeweij, Lidwine W. Tick, Marlène H. W. van de Poel, Otmar R. M. Wikkeling, Louis-Jean Vleming, Ad Koster, Kon-Siong G. Jie, Esther M. G. Jacobs, Nils Planken, Cees H. A. Wittens, Hugo ten Cate, Joachim E. Wildberger, Arina J. ten Cate-Hoek

**Affiliations:** 1 Department of Radiology and Nuclear Medicine, Maastricht University Medical Centre, P.O. Box 5800, 6202 AZ Maastricht, The Netherlands; 2 Department of Radiology and Nuclear Medicine, VieCuri Medical Centre, Venlo, The Netherlands; 3 Department of Vascular Surgery, Maastricht University Medical Centre, Maastricht, The Netherlands; 4 Department of Radiology, Maasstad Hospital, Rotterdam, The Netherlands; 5 Department of Internal Medicine, Maxima Medisch Centrum, Eindhoven, The Netherlands; 6 Department of Internal Medicine, Laurentius Hospital, Roermond, The Netherlands; 7 Department of Vascular Surgery, Heelkunde Friesland Location Nij SMellinghe Hospital, Drachten, The Netherlands; 8 Department of Internal Medicine, Haga Hospital, The Hague, Netherlands; 9 Department of Internal Medicine, VieCuri Medical Centre, Venlo, The Netherlands; 10 Department of Internal Medicine, Zuyderland Medical Centre, Sittard, The Netherlands; 11 Department of Internal Medicine, Elkerliek Hospital, Helmond, The Netherlands; 12 Department of Radiology and Nuclear Medicine, Amsterdam University Medical Centre, location AMC, Amsterdam, The Netherlands; 13 Department of Internal Medicine, Maastricht University Medical Center, Maastricht, The Netherlands; 14 Cardiovascular Research Institute Maastricht (CARIM), Maastricht University, Maastricht, The Netherlands; 15 Center for Thrombosis and Haemostasis, University Medical Center of Gutenberg University, Mainz, Germany; 16 Heart and Vascular Centre and Thrombosis Expertise Centre, Maastricht University Medical Centre, Maastricht, The Netherlands

**Keywords:** Magnetic resonance venography, Thrombosis, Thrombus, Thrombolysis

## Abstract

**Objectives:**

Magnetic resonance venography (MRV) is underutilized in the evaluation of thrombus properties prior to endovascular treatment but may improve procedural outcomes. We therefore investigated the clinical impact of using a dedicated MRV scoring system to assess thrombus characteristics prior to endovascular intervention for iliofemoral deep vein thrombosis (DVT).

**Methods:**

This is a post hoc analysis of data from the CAVA trial (Clinicaltrials.gov:NCT00970619). MRV studies of patients receiving ultrasound-accelerated catheter-directed thrombolysis (CDT) for iliofemoral DVT were reviewed. Thrombus age-related imaging characteristics were scored and translated into an overall score (acute, subacute, or old). MRV scores were compared to patient-reported complaints. MRV-scored groups were compared for CDT duration and success rate.

**Results:**

Fifty-six patients (29 men; age 50.8 ± 16.4 years) were included. Using MRV, 27 thrombi were classified acute, 17 subacute, and 12 old. Based on patient-reported complaints, 11 (91.7%) of these old thrombi would have been categorized acute or subacute, and one (3.7%) of the acute thrombi as old. Average duration of CDT to > 90% restored patency differed significantly between groups (*p *< 0.0001): average duration was 23 h for acute thromboses (range: 19–25), 43 h for subacute (range: 41–62), and 85 h for old thromboses (range: 74–96). CDT was almost eleven times more successful in thromboses characterized as acute and subacute compared to old thromboses (OR: 10.7; 95% CI 2.1–55.5).

**Conclusion:**

A dedicated MRV scoring system can safely discriminate between acute, subacute, and old thromboses. MRV-based selection is predictive of procedural duration and success rate and can help avoid unnecessary complications.

**Key Points:**

• *Thrombus age, characterized by MRV as acute, subacute, and old, can predict CDT duration and probability of success.*

• *Accurate pre-interventional MRV-based thrombus aging has the potential to facilitate identification of eligible patients and may thus prevent CDT-related complications.*

## Introduction

The growing availability of minimally invasive treatment options for deep vein thrombosis (DVT), in particular for iliofemoral DVT, has led to increased use of imaging modalities other than duplex ultrasound in DVT evaluation [1–3]. Accurate pre-interventional imaging of iliofemoral DVT requires evaluation of both abdomino-pelvic and lower extremity veins. In the abdomino-pelvic region, ultrasound is not routinely used or adequate [4, 5]. Both adjunctive magnetic resonance venography (MRV) and computed tomography venography (CTV) have been shown to be feasible. However, CTV has limitations regarding intraluminal changes and beam-hardening artifacts (due to hip replacements for example) and should be avoided in young and pregnant patients. A major disadvantage of CTV is the radiation dose, which is not trivial and should be carefully considered, especially given the oftentimes younger patient population and the need for (long term) repeat examinations [6]. Magnetic resonance venography (MRV) does not require radiation or iodine contrast material and has been shown to be a good option [7–9]. MRV is not only a useful tool for assessing the presence and location of thrombi in the abdomino-pelvic veins, but also for detailed evaluation of thrombus properties. MRV enables identification of several thrombus imaging characteristics [10, 11]. A previous study showed that identifying MRV-specific thrombus characteristics is not only feasible but also reproducible [12]. However, identifying thrombus characteristics is only the first step in utilizing the potential of MRV.

Patients undergoing minimally invasive thrombus removal procedures are at increased risk of thrombolysis-related complications. Therefore, predicting the probability of CDT success prior to treatment is desirable, especially since not all treatments are successful and long-term success depends on adequate primary treatment of acute disease [2, 13, 14]. Being able to predict procedural success could alter preferred treatment strategies. It has previously been shown that MRV imaging characteristics are more accurate than clinical information regarding thrombus age and treatability [15]. To further understand the potential of MRV in iliofemoral DVT, we aim to investigate the relation between treatment outcome and thrombus imaging characteristics on MRV.

The aim of this study was to evaluate if pre-procedural identification of thrombus-age related MRV characteristics of iliofemoral DVT could predict treatment outcomes of catheter-directed thrombolysis (CDT).

## Material and methods

### Patients

This study is a post hoc analysis of the CAVA trial (Clinicaltrials.gov: NCT00970619), an investigator-initiated, multicentre, randomized, single-blind, allocation-concealed, parallel group, superiority trial assessing the development of post-thrombotic syndrome in patients with a first time acute iliofemoral DVT and comparing additional ultrasound-accelerated CDT to standard treatment [3]. The CAVA trial enrolled 184 patients aged 18 to 85 years old, with an objectively documented first-time iliofemoral deep-vein thrombosis (i.e. complete or partial thrombosis of the common femoral vein or more cranial vein segments) with acute symptoms for no longer than 14 days. The patient complaint–based classification included pain and leg swelling as main symptoms and a more detailed analysis using the venous clinical severity score (VCSS). For the full description, we refer to the main trial publication and appendix [3]. Ninety-one of 184 patients were randomly assigned to receive additional ultrasound-accelerated CDT. Fourteen patients did not start the assigned treatment due to early withdrawal (8) or screening failures (6). Therefore, CDT was initiated in 77 patients. Patients allocated to additional ultrasound-accelerated CDT were admitted to a medium care unit at one of the six participating interventional centres, and CDT was started no later than 21 days after onset of patient reported symptoms. The intervention was terminated in case of successful treatment (defined as regained venous patency of > 90% on control angiography, performed every 24h); after 48h treatment without any change in patency on control angiography; in case of persistent fibrinogen levels < 1.8 g/L; or when the maximum duration of treatment (96 h) was reached. Major bleedings were defined as a bleeding associated with a ≥ 2 g/dL fall in haemoglobin, the need for transfusion of two or more units of packed red blood cells or whole blood, a symptomatic bleeding in a critical area or organ (intracranial, intraspinal, intraocular, retroperitoneal, intra-articular, pericardial, or intramuscular), or contributing to the death of the patient [16].

### MRV protocols

All MRV studies of patients in the group receiving additional ultrasound-accelerated CDT of the CAVA trial were reviewed. MRV examinations were performed on clinical MRI systems, based on a master protocol of the principal trial site. The other participating hospitals adapted local scan protocols accordingly. A dedicated 12-element phased-array peripheral vascular coil with a cranio-caudal coverage of 128 cm (Philips Medical Systems) on a 1.5 T MR system (Intera; Philips Medical Systems), was used for signal reception. Patients were imaged in a supine position. An overview of detailed scan parameters is provided in Table 1.

**Table 1:** Scan parameters per participating centre

Hospital	MUMC+	Nijsmellinghe	AMC	Maasstad
Scanner	1.5 T Philips Intera	1.5 T Siemens Magnetom	1.5 T Siemens Magnetom	1.5 T Siemens Magnetom
Sequence	Ultrafast GE	T1 VIBE	T1 VIBE	T1 VIBE
Contrast	Yes	Yes	Yes	Yes
Scan mode	3D	3D	3D	3D
Repetition time (TR) (ms)	7.8	4.73	5.9	3.2
Echo time (TE) (ms)	3.9	2.17	2.44	1.28
Flip angle (degrees)	10	10	20	10
AVG acquisition time (TA) (min)	14:52	12:42	15:39	07:12
Bandwidth (BW) (Hz)	181.8	390	240	521
Acquisition voxel (mm)	0.95 × 0.95 × 3.00	0.91 × 0.91 × 1.80	0.8 × 0.8 × 0.8	1.0 × 1.0 × 6.00
Reconstructed voxel (mm)	0.95 × 0.95 × 1.50	0.91 × 0.91 × 1.80	0.8 × 0.8 × 2.0	0.9 × 0.9 × 1.5
Number of slices	750 (5 × 150)	768 (3 × 256)	537 (3 × 176)	864 (6 × 144)
Acquisition matrix	380 × 266	230 × 256	263 × 350	400 × 313
FoV	400	490	500	400
Fat suppression	Yes	Yes	Yes	Yes
Cardiac synchronization (ECG)	Yes	No	No	Yes
Breath hold	No	No	No	No

Prior to contrast delivery, all patients underwent a standard 2D non-contrast enhanced balanced turbo field echo (BTFE) sequence to visualize the abdominal and pelvic veins. The latter was acquired in 2 volumes to cover the abdomen and pelvis. This was followed by injection of a blood pool contrast agent (Gadofosveset-trisodium, Ablavar, Lantheus Medical Imaging). A fixed dose of 10 mL Gadofosveset-trisodium (0.25 mmol/mL) was administered intravenously at 1 mL/s in the median cubital vein, followed by a 20 mL saline flush injected at the same flow rate, using a remote-controlled dual head injector (Spectris; Bayer Medrad). Acquisition of the first scan volume was started 30 s after contrast administration.

A three-dimensional (3D) ultrafast gradient echo sequence with fat suppression (spectral presaturation with inversion recovery, SPIR) was used for high-resolution imaging of the venous vasculature. Coverage of the deep vein system from the IVC to the distal popliteal vein/proximal calf veins was ensured by a coronal acquisition scheme in 3 volumes covering abdomen, pelvis, and (upper) legs, which were then stitched and reconstructed in the axial plane on the scanner.

### Angiography and catheter-directed thrombolysis

Routinely, the deep vein system was accessed from the popliteal vein and contrast angiography was performed from the popliteal vein to the inferior caval vein. After positioning of the thrombolysis catheter in the thrombotic occlusion, thrombolytic treatment was started (T0). Control angiography was performed every 24 h [3].

### Evaluation of imaging studies

Thrombus age-related imaging characteristics were scored for the common femoral vein of the affected limb. The following items were subjectively scored: image quality, confidence of image interpretation, and thrombus characteristics.

Image quality was assessed subjectively on a 5-point scale modified from Danias et al: 1 poor-quality information, non-diagnostic; 2 structures visible but with significant blurring/artifacts, diagnosis suspected but not established; 3 anatomy visible with moderate blurring/artifacts, able to establish diagnosis; 4 minimal blurring/artifacts, good-quality diagnostic information with definite diagnosis; and 5 sharply defined borders, excellent quality diagnostic information [17, 18].

*Confidence of image interpretation* was scored on a scale from 1 to 4, with 1 = unsure (definite interpretation unsure), 2 = mildly confident (evaluation of major findings possible), 3 = moderately confident (definite interpretation possible), and 4 = confident (exact interpretation possible) [17–19].

**Fig. 1:**
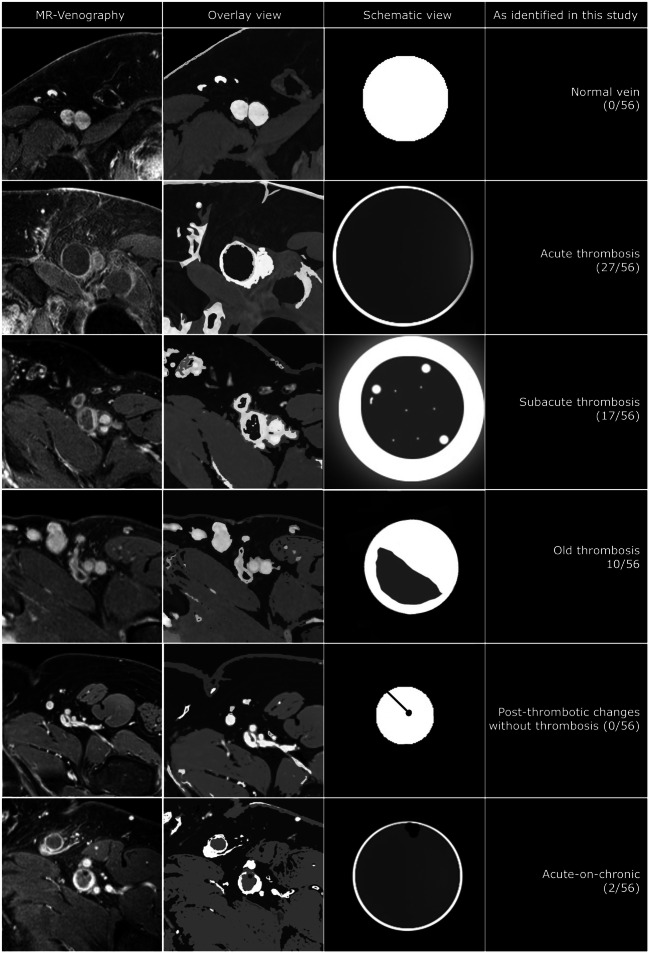
Thrombus characteristics identified using MR-venography. Normal vein: homogeneously opacified hyperintense vein lumen. No luminal defect or perivascular) wall changes. Acute thrombosed vein: dilated homogeneously hypointense vein lumen with small enhancing rim of contrast depicting the vein wall. No (perivascular) wall changes (no halo sign). Subacute thrombosed vein: Still dilated low intensity vein lumen with thick enhancing rim of contrast, part vein wall thickening and part perivascular edema (halo sign). There are some small hyperintense areas within the thrombus as sign of recanalization. Old thrombosed vein: the vein lumen is reduced to a more ‘normal’ vein size with an opacified part (open lumen/vein wall) and a low intensity part that is still filled with thrombus-like tissue. Post-thrombotic vein: the vein lumen is smaller than the normal vein and homogeneously opacified except for 1 or more sharply demarcated very low intensity black dots and/or lines adhering to the vein wall. This represents (fibrotic) scar tissue (post-thrombotic venous scarring). Acute-on-chronic thrombosed vein: as in an acute deep vein thrombosis there is a dilated lumen with mostly hypointense material but additionally there are signs of a previous thrombotic event that has left scar tissue markings (very hypointense dots and lines)

*Thrombus age-related imaging characteristics* were based on a previously developed and validated scoring system [12], described as dilatation of the vein (increased size), hypointense signal intensity within the vein lumen, signs of recanalization, presence of wall thickening with a halo sign, or post-thrombotic scarring (Fig. 1). Finally, an overall thrombus score was assigned. Interobserver agreement for the identification of thrombus characteristics based on this scoring system was previously reported to be excellent between expert radiologist (k 0.97) and good for novice radiologists (k 0.82) [12].

A dilated vein with hypointense signal intensity was assigned the overall score ‘acute’. A dilated vein showing wall thickening, the halo sign, hypointense signal intensity, and signs of recanalization was scored as ‘subacute’. A non-dilated vein showing a (partial) hypointense signal intensity with or without signs of post-thrombotic scarring and wall thickening was labelled as ‘old’. A dilated vein with hypointense signal intensity and additionally signs of post-thrombotic scarring (possibly so-called acute-on-chronic characteristics) was also labelled as ‘old’.

All sequences for this study were evaluated by 2 independent reviewers (CA, cardiovascular and interventional radiologist with 11 years of expertise, and RB, interventional radiologist with 7 years of expertise). The reviewers had access to source images as well as common post-processing tools (MPR/curved planar reconstruction, MIP). In patients with a DVT on both sides, whether both sides were intervened on was left to the treating specialist‘s discretion. Only the most severely diseased side (clinically) was evaluated in the CAVA analysis. The reviewers were informed whether the left or right side was to be evaluated, but otherwise blinded for duplex ultrasound findings and clinical records of the patients. After independently reviewing the images, consensus was reached for all cases between the reviewers. These outcomes were used for the overall statistical analysis.

### Statistical analysis

To compare outcomes for continuous variables between groups, one-way analysis of variance (ANOVA) or Kruskal-Wallis was used, as appropriate. In case of overall significant findings, pairwise comparisons were examined using Tukey post hoc adjustment or Mann-Whitney *U* test, as appropriate.

To assess the difference in proportions, univariate analysis with logistic regression (chi-square) was used, and associated odds ratios (ORs) with corresponding 95% confidence intervals (95% CIs) were calculated.

Interobserver agreement was calculated using the kappa statistic.

For all analyses, a *p* - value of < 0.05 was considered statistically significant. The statistical analyses were performed using SPSS, version 24 (IBM corporation).

## Results

### Patients

**Fig. 2:**
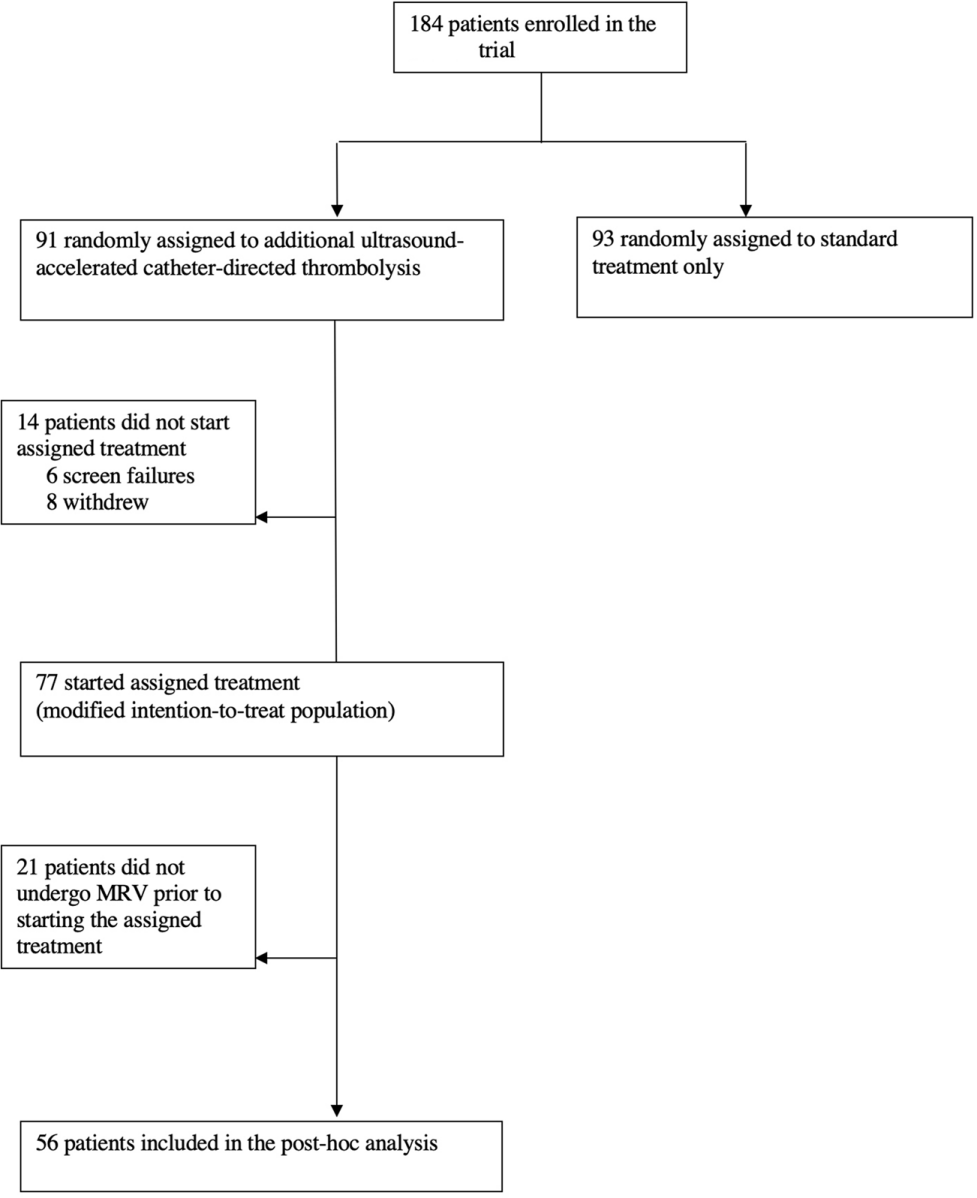
Trial profile

Figure 2 shows the inclusion profile for this post hoc analysis of CAVA study data. Twenty-one of the 77 patients receiving additional ultrasound-accelerated CDT in the CAVA study did not undergo MRV prior to the start of the procedure due to logistic reasons and were excluded from this analysis, leaving a total of 56 patients available for inclusion.

### Image quality

Overall MRV image quality was rated as excellent with an average score of 4.61 ± 0.59. Confidence of image interpretation was high with an average score of 3.86 ± 0.35.

### Interobserver agreement

Overall interobserver agreement was excellent with a kappa of 0.85, confirming the previously reported high level of agreement between observers [12].

### Thrombus age-related MRV-imaging characteristics

Distribution of the six thrombus age-related imaging elements in relation to the overall scores (acute, subacute, and old) is shown in Table 2. Dilated vein segments were significantly more often present in acute and subacute thrombosis. Signs of recanalization were most often present in subacute thrombosis), as was a halo sign around the vein. Partial very hypointense vein lumen was only present in old thrombosis as were all but 1 sign of post-thrombotic scarring of the vein wall.

**Table 2:** Overall thrombus age in relation to imaging characteristics

	Acute *N* = 27	Subacute *N* = 17	Old *N* = 12	Total *N* = 56
Hypointense signal intensity vein lumen	27 (100.0%)	17 (100.0%)	12 (100.0%)	56 (100.0%)
Dilated vein^*^	27 (100.0%)	16 (94.1%)	3 (25.0%)	46 (82.1%)
Signs of recanalization^*^	2 (7.4%)	17 (100.0%)	9 (75.0%)	28 (50.0%)
Thickened vein wall with halo sign around vein^*^	0	17 (100.0%)	4 (33.3%)	21 (37.5%)
Partial very hypointense vein lumen^*^	0	0	10 (83.3%)	10 (17.9%)
Post-thrombotic scarring^†^	0	1 (5.9%)	4 (33.3%)	5 (8.9%)

The above table shows the scores of individual thrombus characteristics. Data are *n* (%). ^*^p < 0.0001, ^†^*p* = 0.007

### Overall thrombus score

**Fig. 3:**
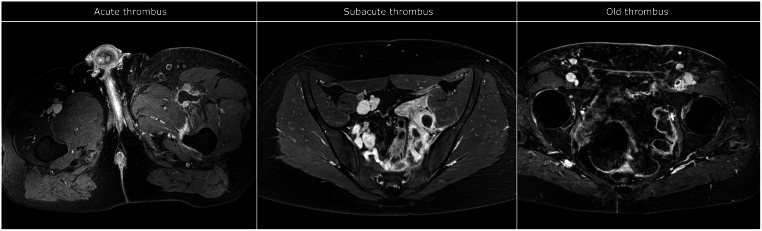
Examples of variations in characteristics of iliofemoral DVT. From left to right: examples of left-sided acute common femoral, subacute iliac and old femoro-iliac thrombi as identified in the studies examined. Notice how the acute case shows a very homogenous ‘clean’ image with subcutaneous edema. In contrast, there is extensive perivascular edema in the subacute image and more inhomogeneous signal intensities in the old image

Of the 56 cases evaluated, 27 were characterized as acute thrombus, 17 as subacute thrombus, and 12 as old thrombus. Examples are shown in Fig. 3. The old group included 3 cases with so-called mixed characteristics. They were described as showing ‘acute-on-chronic’ thrombosis implying a re-thrombosis of the affected iliofemoral vein(s), showing both post-thrombotic scarring and dilation of a vein filled with hypointense signal.

### Patient and treatment characteristics versus overall thrombus score

Table 3 shows patient and treatment characteristics stratified per thrombus-age group, as determined using MRV.

**Table 3:** Patient characteristics per MRV-based thrombus age group

	Acute *N* = 27	Subacute *N* = 17	Old *N* = 12	Total *N* = 56
Age, years—mean ± SD	51.7 ± 16.0	54.1 ± 17.5	44.1 ± 15.2	50.8 ± 16.4
Age, years—categories				
- < 40 years - 40 – 65 years - > 65 years	6 (22.2%) 15 (55.6%) 6 (22.2%)	4 (23.5%) 6 (35.3%) 7 (41.2%)	4 (33.3%) 6 (50.0%) 2 (16.7%)	14 (25.0%) 27 (48.2%) 15 (26.8%)
sex, male	16 (59.3%)	8 (47.1%)	5 (41.7%)	29 (51.8%)
Affected side^*^				
- Left ^†^ - Right ^†^ - Bothsided	18 (66.7%) 8 (29.6%) 1 (3.7%)	17 (100.0%) 0 0	3 (25.0%) 8 (66.7%) 1 (8.3%)	38 (67.9%) 16 (28.6%) 2 (3.6%)
BMI—mean ± SD	28.1 ± 5.9	28.2 ± 3.7	29.2 ± 8.3	28.4 ± 5.9
BMI, categories				
- < 25.0 - 25.0 – 29.9 - ≥ 30.0 - Unknown	9 (33.3%) 10 (37.0%) 7 (25.9%) 1 (3.7%)	4 (23.5%) 8 (47.1%) 5 (29.4%) 0	4 (33.3%) 5 (41.7%) 3 (25.0%) 0	17 (30.4%) 23 (41.1%) 15 (26.8%) 1 (1.8%)
Duration of complaints at MRV imaging, days—mean ± SD^‡^	8.5 ± 4.5	9.8 ± 5.5	14.0 ± 4.2	10.1 ± 5.2
Duration between MRV and start thrombolysis, days—mean ± SD	0.96 ± 2.0	1.59 ± 2.7	1.08 ± 1.93	1.2 ± 2.2
Duration of complaints at start thrombolysis, days—mean ± SD^§^	9.5 ± 5.1	11.4 ± 4.9	15.1 ± 4.0	11.3 ± 5.2
Duration of complaints at start thrombolysis, categories				
- 0 – 7 days - 7 – 14 days - 14 – 21 days ^¶^ - > 21 days - Unknown	9 (33.3%) 12 (44.4%) 4 (14.8%) 1 (3.7%) 1 (3.7%)	2 (11.8%) 9 (52.9%) 6 (35.3%) 0 0	0 4 (33.3%) 7 (58.3%) 1 (8.3%) 0	11 (19.6%) 25 (44.6%) 17 (30.4%) 2 (3.6%) 1 (1.8%)
Successful thrombolysis^§^	19 (70.4%)	11 (64.7%)	2 (16.7%)	32 (57.1%)
Total time of thrombolysis, h—mean ± SD^†^	23.3 ± 7.4	47.9 ± 19.3	85.3 ± 16.3	44.1 ± 27.8
Complications, major bleeding	1 (3.7%)	2 (11.8%)	0	3 (5.4%)

Data are *n* (%) or mean (SD)

^*^p = 0.002, ^†^*p* = 0.000, ^‡^*p* = 0.007, ^§^*p* = 0.006, ^¶^*p* = 0.026 ^ǁ^

The average thrombolysis time was 23 (19–25) hours for acute, 43 (41–62) hours for subacute, and 85 (74–96) hours for old thrombi (*p* < 0.0001).

Thrombolysis was successful in 32 of 56 patients (57.1%) and was more often successful in combined acute and subacute thrombosis groups, with 30 of 44 (68.2%) successful interventions, than in the old group, with 2 of 12 successful interventions (16.7%) (OR = 10.71 (2.07–55.5), *p *= 0.006). Ten out of 12 thrombolysis procedures in the old thrombus group were unsuccessful, either due to premature termination or incomplete lysis at 96 h (per protocol maximum duration of thrombolytic therapy).

Of the 12 thrombi categorized as old using MRV assessment, 11 (91.7%) were categorized as acute or subacute based on patient-reported duration of complaints. On the other hand, one (3.7%) of the thrombi classified as acute using MRV was categorized as old based on patient-reported complaints. Treatment of the thrombus with CDT in this case (clinically ‘old’, MRV ‘acute’) was successful.

Three cases of major bleeding occurred (5,4%): one in a patient with acute thrombosis, two in patients with subacute thromboses, and none in patients with old thromboses.

## Discussion

The results of this study show that MRV may be a useful diagnostic modality for assessing thrombus age-related characteristics prior to CDT in iliofemoral DVT. Using MRV, thromboses could be identified as acute, subacute, and old in all cases. There was a clear discrepancy between patient complaint–based classification and MRV-based classification, in particular in the old thromboses group. Moreover, MRV-estimated thrombus age was found to be associated with both the duration and success rate of the intervention. The average thrombolysis time significantly differed between MRV-based groups with favourable results (shorter thrombolysis times and higher procedural success rate) in the acute and subacute versus the old thromboses group.

MRV therefore enables a pre-selection of patients who are most likely to benefit from CDT, and those for whom the better option is to withhold thrombolysis (i.e. in cases where old thrombus age predicts poor success rate). The latter patients, being exposed to the risk of thrombolytic therapy with little to no benefit regarding thrombus removal, potentially stand to gain most from this pre-interventional assessment.

It is generally accepted that thrombi older than 21 days are resistant to thrombolytic therapy [1, 2]. The current findings show that MRV-based thrombus age is almost eleven times more likely to be accurate than thrombus age based on patient complaints. The latter was inaccurate in 21% of cases, most of which failed to reach adequate recanalization following thrombolytic therapy. This might explain the relatively high failure rate of the CDT found in the CAVA trial [3]. Had thrombolysis been withheld in patients with a thrombus characterized as old on MRV in this series, the overall procedural success rate could have increased by 11%.

No benefit concerning procedural related bleeding complications could be identified in this series, but this may be due to the limited number of adverse events in the CAVA trial.

Unexpectedly, signs of a previous DVT event with remnant scarring within the femoral veins were found in three patients with acute-on-chronic thrombus characteristics. These vein wall changes are common in post-thrombotic deep vein disease but were not anticipated in patients with first-time acute DVT. Clinically, these previous thrombotic events were asymptomatic and had not been identified during patient intake, emphasizing the need for a better diagnostic work-up before proceeding to CDT.

There is an evolution of the thrombus characteristics over time and these characteristics are clearly distinctive. However, there are gradual changes when the thrombus evolves from the acute to the subacute and later old phase in iliofemoral DVT cases: in addition to the well-defined criteria of the described scoring system, the presence and extent of not only perivascular but also subcutaneous edema were observed. In the acute phase, the latter can be very extensive. In the subacute phase, edema was still present but tended to organize more around the vein (wall) as perivascular edema. In the old phase, the (visible) edema was mostly resolved. While the confidence of image interpretation with the imaging features studied was already excellent, assessment of subcutaneous edema could potentially be an additional visual aid for radiologists starting to assign a thrombus score in daily practice. Extensive venous collateralization is generally a sign of a more chronic venous occlusive state, and should not be present in acute DVT [19]. It was not observed in any of the cases in this study.

However, there are some limitations to this study. First, this is a post hoc analysis with a small sample size. However, distinctions between the three categories of MVR-based thrombus age are clear and associations with clinical outcomes strong. A second limitation is that the MRV scan protocol for the CAVA trial included the blood pool contrast agent gadofosveset-trisodium, the use of which is no longer current daily practice. Studies have shown the benefits of using a standard extracellular gadolinium agent [20, 21]. The blood pool contrast agent can be substituted with a standard extracellular gadolinium agent containing gadobutrol (Gadovist, Bayer HealthCare) without loss of image quality or diagnostic value. This standard agent is now routinely administered in our practice and has replaced blood pool contrast agents for MRV [22]. Third, although widely used, patient-reported complaints are not a robust indicator for exact thrombus age [15]; in fact, a partial thrombosis may last for a prolonged period of time before becoming occlusive and therefore symptomatic [23]. In this study, MRV was shown to provide a more objective and robust indication for thrombus age than patient-reported complaints. Fourth, CDT was not successful in all acute thromboses and ultimate success of reperfusion therapy may not rely solely on adequate assessment of thrombus age. Other patient characteristics and properties of the thrombus which influence clot resolution may be important additional determinants. For example, differences in endogenous clot lysis due to individual patient variation in clot structures influence turbidity and permeation and are independent of thrombus age [24].

In conclusion, thrombus aging based on MRV imaging enables preprocedural selection of patients with iliofemoral DVT most likely to undergo successful CDT, as well as those most likely to be resistant to thrombolytic therapy. This helps to avoid unnecessary risk associated with unsuccessful catheter-directed thrombolysis and extensive treatment duration. In view of these results, magnetic resonance venography should be considered a prerequisite for patients opting to undergo catheter-directed thrombolysis for iliofemoral deep vein thrombosis.

## Summary statement

Pre-interventional magnetic resonance venography–based assessment of thrombus age in patients with iliofemoral deep vein thrombosis can identify patients most likely to undergo successful catheter-directed thrombolysis and may thus prevent unnecessary catheter-directed thrombolysis-related complications.

## Abbreviations and acronyms

CDT: Catheter-directed thrombolysis
DVT: Deep vein thrombosis
MRV: Magnetic resonance venography

## Declarations

### Guarantor

The scientific guarantor of this publication is C.W.K.P. Arnoldussen.

### Statistics and biometry

Two of the authors have significant statistical expertise (P. Notten, A.J. ten Cate-Hoek). No complex statistical methods were necessary for this paper.

### Study subjects or cohorts overlap

This is a post hoc analysis of the CAVA trial. Ultrasound-accelerated catheter-directed thrombolysis versus anticoagulation for the prevention of post-thrombotic syndrome: a single-blind, multicentre, randomised trial [3].

### Methodology

• Prospective

• Diagnostic or prognostic study

• multicentre study
